# Identification and characterization of a solute carrier, CIA8, involved in inorganic carbon acclimation in *Chlamydomonas reinhardtii*

**DOI:** 10.1093/jxb/erx189

**Published:** 2017-06-13

**Authors:** Marylou C Machingura, Joanna Bajsa-Hirschel, Susan M Laborde, Joshua B Schwartzenburg, Bratati Mukherjee, Ananya Mukherjee, Steve V Pollock, Britta Förster, G Dean Price, James V Moroney

**Affiliations:** 1 Department of Biological Sciences, Louisiana State University, Baton Rouge, LA, USA; 2 ARC Centre of Excellence for Translational Photosynthesis, Division of Plant Sciences, Research School of Biology, Australian National University, Canberra ACT, Australia

**Keywords:** Bile acid transporter, CCM, *Chlamydomonas reinhardtii*, CO_2_ assimilation, inorganic carbon uptake

## Abstract

The supply of inorganic carbon (Ci) at the site of fixation by Rubisco is a key parameter for efficient CO_2_ fixation in aquatic organisms including the green alga, *Chlamydomonas reinhardtii*. *Chlamydomonas reinhardtii* cells, when grown on limiting CO_2_, have a CO_2_-concentrating mechanism (CCM) that functions to concentrate CO_2_ at the site of Rubisco. Proteins thought to be involved in inorganic carbon uptake have been identified and localized to the plasma membrane or chloroplast envelope. However, current CCM models suggest that additional molecular components are involved in Ci uptake. In this study, the gene *Cia8* was identified in an insertional mutagenesis screen and characterized. The protein encoded by *Cia8* belongs to the sodium bile acid symporter subfamily. Transcript levels for this gene were significantly up-regulated when the cells were grown on low CO_2_. The c*ia8* mutant exhibited reduced growth and reduced affinity for Ci when grown in limiting CO_2_ conditions. Prediction programs localize this protein to the chloroplast. Ci uptake and the photosynthetic rate, particularly at high external pH, were reduced in the mutant. The results are consistent with the model that CIA8 is involved in Ci uptake in *C. reinhardtii*.

## Teaser

CIA8 is involved in bicarbonate uptake in the carbon dioxide-concentrating mechanism (CCM) of *Chlamydomonas reinhardtii*.

## Introduction

Green algae and other photosynthetic aquatic organisms are often exposed to low and fluctuating CO_2_ conditions in the natural environment. CO_2_ availability for these organisms can be restricted by, among other factors: slow diffusion of gases in water, slow interconversion of the two inorganic carbon (Ci) forms (CO_2_ and HCO_3_^–^), and pH changes. Consequently, almost all unicellular aquatic photosynthetic organisms have evolved a CO_2_-concentrating mechanism (CCM) inducible under limiting CO_2_ conditions, to concentrate Ci effectively for fixation by Rubisco (Giordano *et al.*, 2005). In a current CCM model for the green alga *Chlamydomonas reinhardtii* (Jungnick *et al.*, 2014; Wang and Spalding, 2014) it is thought that bicarbonate transporters on the plasma membrane and chloroplast envelope are key components of the CCM allowing the movement of Ci, particularly HCO_3_^–^, through the membranes. Other CCM components include carbonic anhydrase enzymes that interconvert CO_2_ and HCO_3_^–^ (Mitra *et al.*, 2005; Moroney *et al.*, 2011), and a prominent compartment called the pyrenoid which is a dense protein complex in the chloroplast. The pyrenoid is where Rubisco is sequestered under limiting CO_2_ conditions (Kuchitsu *et al.*, 1988; Rawat *et al.*, 1996; Borkhsenious *et al.*, 1998; Mackinder *et al.*, 2016). An extensive network of thylakoid tubules and mini-tubules is associated with the pyrenoid (Engel *et al.*, 2015; Mackinder *et al.*, 2016) presumably to provide a pathway for HCO_3_^–^ to enter into the pyrenoid. The carbonic anhydrase, CAH3, is found in these tubules and it is hypothesized that CAH3 converts the HCO_3_^–^ within the lumen to CO_2_ for fixation (Karlsson *et al.*, 1998; Moroney and Ynalvez, 2007; Blanco-Rivero *et al.*, 2012).

*Chlamydomonas reinhardtii* cells grown under high CO_2_ conditions (5% v/v in air) exhibit a low affinity for Ci. When high-CO_2_-acclimated cells are exposed to low CO_2_ conditions (0.04% v/v), induction of high affinity transporters has been reported. While CO_2_ will readily diffuse across membranes in the cell (Gutknecht *et al.*, 1977), numerous studies have since established the need for an active transport system to facilitate the movement of Ci (particularly HCO_3_^–^) to the point of fixation by Rubisco in low CO_2_ cells (Moroney *et al.*, 1987; Sültemeyer *et al.*, 1988; Badger *et al.*, 1994; Ohnishi *et al.*, 2010). In addition, molecular and physiological studies have also confirmed the occurrence of multiple forms of Ci transporters on the plasma membrane and chloroplast envelope of microalgal cells (Amoroso *et al.*, 1998; Duanmu *et al.*, 2009; Atkinson *et al.*, 2015; Gao *et al.*, 2015; Yamano *et al.*, 2015). In marine cyanobacteria, HCO_3_^–^ transport at the plasma membrane is often coupled to the external Na^+^ ion concentration (Price *et al.*, 2008). In freshwater environments where *C. reinhardtii* is found, transport is thought to be H^+^ coupled since Na^+^ is relatively low (Morth *et al.*, 2011; Taylor *et al.*, 2012). Consequently, genomic studies with *C. reinhardtii* and *Volvox carteri* have revealed the presence of both H^+^- and Na^+^-coupled transporters at least for sulphate and phosphate (Pootakham *et al.*, 2010). It is not yet clear whether these ions are also needed for bicarbonate uptake in *C. reinhardtii*.

To date, one low and two high affinity bicarbonate transport proteins in *C. reinhardtii* are characterized and known to be functional under low CO_2_ conditions. The first, a high-light-activated protein (HLA3) is an ATP-binding cassette (ABC)-type transporter of the Multi-Drug Resistance protein family localized to the plasma membrane (Im and Grossman, 2002). The *Hla3* transcript is induced by both high light and low CO_2_ conditions and is controlled by the CCM1 ‘master regulator’ encoded by the *Cia5* gene. Duanmu *et al.* (2009) showed in HLA3 RNAi knockdown mutants, a significant reduction in Ci affinity and Ci uptake, supporting the role of the protein in HCO_3_^–^ transport. The second, the novel transporter LCI1, is a relatively small protein with little homology to other transmembrane proteins in the databases. LCI1 is strongly up-regulated under low CO_2_ conditions and has been localized to the plasma membrane (Ohnishi *et al.*, 2010). In this study with LCI1, the authors also confirmed increased Ci uptake by overexpressing LCI1 protein in the *Lcr1* (*C. reinhardtii* strain lacking a MYB transcription factor) background (Ohnishi *et al.*, 2010). Thus, HLA3 and LCI1 are thought to be Ci transporters located on the plasma membrane.

The third transporter, NAR1.2 (also known as LCIA), is a chloroplast envelope protein of the Formate/Nitrite transporter family. Although the NAR1.2 protein has lower affinity for bicarbonate as revealed by the *K*_(0.5)_ value which falls in the low millimolar range, a modest increase in HCO_3_^–^ uptake is observed in *Xenopus laevis* oocytes when NAR1.2 is expressed in those cells (Mariscal *et al.*, 2006; Atkinson *et al.*, 2015). NAR1.2 has so far been attributed to Ci uptake on the chloroplast envelope even though there has been no direct evidence to support this. While the NAR1.2 protein plays an important role in Ci uptake, it may also have a regulatory function. This follows from a recent study in which *Hla3* transcript did not accumulate in the absence of NAR1.2 protein; hence, the authors suggest that these proteins co-operate in bicarbonate accumulation (Yamano *et al.*, 2015). In another proposed bicarbonate route into the chloroplast, NAR1.2 seems also to associate with a soluble protein LCIB (Wang and Spalding, 2014).

Two soluble proteins (LCIB/LCIC) form a complex, and are thought to be involved in recapture of CO_2_ leaking from the pyrenoid after observations that they closely associate with the pyrenoid when cells are acclimated to low CO_2_ (Yamano *et al.*, 2010; Wang *et al.*, 2011). Other putative transporters CCP1 and CCP2, now confirmed to be mitochondrial (Atkinson *et al.*, 2015), are yet to be resolved. In light of all this, it is evident that the Ci transport system in *C. reinhardtii* remains to be further clarified in order to have a better understanding of the CCM.

In this study, we report for the first time the identification and characterization of a gene, which we designate *Cia8* (Phytozome ID: Cre09.g395700), encoding a solute carrier protein, identified in a mutagenesis screen. The *Cia8* gene encodes a transmembrane protein that belongs to the Na^+^/bile acid symporter family (SBF/BASS) also referred to as SLC10, a group of conserved transmembrane proteins (Pushkin and Kurtz, 2006). Our results show that the *Cia8* gene product is needed for optimal growth on low CO_2_ and appears to be needed for bicarbonate uptake in *C. reinhardtii*.

## Materials and methods

### Cell culture and growth

*Chlamydomonas reinhardtii* culture conditions were the same as described in Ma *et al.* (2011). The D66 strain (*nit2*^–^*, cw15, mt*^*+*^) was obtained from Dr Rogene Schnell (University of Arkansas, Little Rock) and the cc124 strain (*nit1*^–^*, nit2*^–^*, mt*^–^) was obtained from the Chlamydomonas Genetics Center at Duke University (http://www.chlamy.org/). Tris-acetate-phosphate pH 7.3 (TAP) and minimal pH 6.8 (MIN) media (without acetate) were prepared according to Sueoka (1960). Both TAP and MIN plates for the growth medium were prepared by adding 1.2% (w/v) agar. Cell cultures were initiated by inoculating colonies from TAP plates into 100 ml of TAP liquid medium in Erlenmeyer flasks. Cultures were grown to early log phase with continuous illumination (100 µmol m^–2^ s^–1^) and shaking for 48 h. The TAP-grown cultures were harvested and washed with MIN medium, and re-suspended in MIN medium, connected to high CO_2_ (5% v/v CO_2_ in air) bubbling for 48 h to reach a cell density OD_730_ of between 0.2 and 0.3 (~2–3 × 10^6^ cells ml^–1^). For CCM induction, the cells were transferred to low CO_2_ (0.01% v/v CO_2_ in air) bubbling for 4 h.

### Mutagenesis, isolation of growth impaired CO_2_ mutants, and phenotypic screen

Mutants were generated in a mutagenesis screen according to Jungnick *et al.* (2014). A linear plasmid (pSL18) bearing the *AphVIII* gene conferring paromomycin resistance (para^R^) was transformed into the D66 strain of *C. reinhardtii* by electroporation (Shimogawara *et al.*, 1998). Transformed cells were selected on TAP plates containing the antibiotic paromomycin (7.5 µg ml^–1^; Invitrogen). Antibiotic-resistant strains were screened for growth in a high CO_2_ chamber (5% v/v CO_2_ in air) and a low CO_2_ chamber (0.01% v/v CO_2_ in air) with the same light conditions as mentioned above. Cells were streaked on MIN plates and placed in growth chambers. Spot tests were done by suspending actively growing cells in liquid MIN medium to the same cell densities (OD_730_ =0.15, 0.07, and 0.03) and spotting 15 µl of each suspension onto MIN plates. These were placed in high, ambient, and low CO_2_ chambers for 7 d. CO_2_ concentration in the growth chambers was measured using an Environmental Gas Monitor (EGM-4, PP systems, Amesbury, MA, USA).

### Identification of the flanking region

An adaptor-mediated PCR method was used to identify the DNA region flanking the *AphVIII* insertion (Pollock *et al.*, 2017). Homology searches were done using the Joint Genome Institute *C. reinhardtii* in the Phytozome database version 10.3: https://phytozome.jgi.doe.gov/ (Merchant *et al.*, 2007; Blaby *et al.*, 2014).

### Linkage analysis

Genetic crosses and tetrad analysis were done as described previously (Moroney *et al.*, 1986; Harris, 2009). Briefly, c*ia8* (mt^+^) and *cc124* (mt^–^) cell cultures in log phase were transferred to nitrogen-deficient TAP medium in the light overnight to induce gametogenesis. The next morning, 3 ml of each culture were mixed to allow mating for 3 h in the light. Aliquots (0.5 ml) were plated on TAP minus-nitrogen medium containing 4% agar. The plates were stored in the dark for 2 weeks to allow zygote maturation. After 14 d, zygotes were transferred to 1.2% agar TAP medium plates for meiotic germination. Tetrad dissections were conducted and linkage determined by association of the paromomycin resistance gene with progeny that did not grow well on low CO_2_.

### Photosynthetic assays

*Chlamydomonas reinhardtii* cultures were started heterotrophically in 100 ml of TAP medium for 48 h to reach log phase. The cells were centrifuged and transferred to 250 ml Erlenmeyer flasks and re-suspended in MIN medium, bubbled with 5% CO_2_ until they reached a cell density OD_730_=0.2–0.3 (3 × 10^6^ cells ml^–1^). The cultures were then transferred to low CO_2_ (0.01%) for 4 h to allow CCM induction. The affinity for external Ci (*K*_0.5_[Ci]) was estimated according to Ma *et al.* (2011). In the method, cells with an equivalent of 100 µg of chlorophyll were suspended in 25 mM HEPES-KOH buffer (pH 7.3) or 25 mM CHES-KOH buffer (pH 9.0) bubbled with inert nitrogen gas (i.e CO_2_ free). The cells were transferred to an O_2_ electrode chamber (Rank Brothers, Cambridge, UK) illuminated at 300 µmol m^–2^ s^–1^, and left to deplete any remaining Ci in the buffer and intracellular spaces. Upon depletion of endogenous CO_2_, no net O_2_ evolution is observed. Known concentrations of NaHCO_3_ were injected into the chamber and the rate of O_2_ evolution was measured. The *K*_0.5_[Ci] was calculated as the Ci concentration required for half-maximal rates of oxygen evolution (Badger, 1985). Chlorophyll content was measured by combining Chl *a* and *b*. Chlorophyll was extracted in 100% methanol and measured using a spectrophotometer. The *K*_0.5_ (CO_2_) is taken as the CO_2_ concentration needed to reach half *V*_max_ O_2_ evolution.

### Intracellular localization

The whole Cre09.395700 gene (4846 bp) was amplified and introduced into a modified pSL18CrGFP vector using *Eco*RI and *Nde*I restriction sites. The Cre09.395700 gene was inserted such that it was in-frame with the *Chlamydomonas* codon-optimized CrGFP (*C. reinhardtii* green fluorescent protein) gene (Fuhrmann *et al.*, 1999) already in the vector. The vector was linearized with *Kpn*I digestion. Transformation of the wild-type strain D66 with the *Cia8* gene fragment was achieved by electroporation with the linearized vector, and paromomycin-resistant colonies were analysed using PCR. For imaging CrGFP-tagged proteins, 5 μl of cells were mounted onto a slide with 1.5% low melting point agarose. Cells were imaged using a Leica Sp2 confocal microscope. The Kr/Ar laser was set at a wavelength of 488 nm to excite both CrGFP and chlorophyll, with the photomultiplier tube set to 500–520 nm to detect GFP fluorescence and 660–700 nm to detect chlorophyll autofluorescence. A ×20 lens was used to image the cells.

### Gene expression analysis

RNA extraction was done using Triazol reagent following the guidelines provided (Invitrogen, Carlsbad, CA, USA). A 1 µg aliquot of total RNA was used as template for synthesis of cDNA. The Superscript First Strand Synthesis System for transcripts with high GC content (Invitrogen) was used to synthesize cDNA according to the manufacturer’s instructions. An aliquot of 100 ng of cDNA was used as the template with SYBR Select (Applied Biosystems, Foster City, CA, USA) for quantitative PCR in an ABI Prism 7000 sequence detection system following the manufacturer’s instructions (Applied Biosystems). Normalized primers were specific for the glyceraldehyde phosphate dehydrogenase (GAPDH) gene for quantitative real-time PCR (qPCR). For qPCR, the *Cblp* gene was used as a control (Schloss, 1990). The primers used for the respective cDNAs are listed in Supplementary *Table S1* at JXB online.

### *Complementation of* Cia8

Complementation of the *cia8* mutant was achieved by transformation of *cia8* cells with a construct containing a wild-type copy of the *Cia8* gene. The construct consisted of the *Cia8* coding sequence (CDS) fragment including the 5'- and 3'-untranslated regions (UTRs; 2949 bp) and a 1080 bp fragment of the promoter region. These fragments were ligated using the *Not*I restriction site, which was then ligated into pGEM-T. The sequenced fragment was cloned into the shuttle vector pSP124s using *Bam*HI and *Sac*I restriction sites. Sequences were obtained from Phytozome Version 10.2 (http://www.phytozome.net/). Primers used to amplify the *Cia8* gene fragments are shown in Supplementary Table S1. The electroporation method was used for cell transformation, and strains were selected for bleomycin resistance (Shimogawara *et al.*, 1998). The presence of complemented DNA in selected *Chlamydomonas* strains was confirmed by PCR.

### Ci uptake assay

Active species uptake of H^14^CO_3_^–^ was carried out at pH 9.0 (25 °C) with 15, 60, and 240 s uptake periods, terminated by silicone oil centrifugation–filtration principally following the protocol of Badger *et al.* (1980). Cells acclimated to high and low CO_2_ conditions were harvested and resuspended at 16 µg of chlorophyll ml^–1^ in 50 mM HEPES-NaOH, pH 7.8, and illuminated (300 μmol m^–2^ s^–1^ white light) to deplete endogenous Ci. Cells were then harvested and resuspended in the same volume of 50 mM HEPES-NaOH, pH 9, and 200 µl aliquots of cell suspension were layered on top of the silicon oil in 400 µl microfuge tubes in the light. A radioactive 25 mM bicarbonate solution (pH ~9.5) was made by mixing radioactive NaH^14^CO_3_ and NaHCO_3_. Uptake measurements were initiated by adding radioactive bicarbonate to a final concentration of 500 μM to the cells in the light. After centrifugation, each cell pellet was resuspended in 200 µl of 2 N NaOH and divided into two 100 µl aliquots. One aliquot was used to determine total NaHCO_3_ taken up, whereas the other was acidified with HCl to determine the proportion of photosynthetically fixed CO_2_. Intracellular bicarbonate pool sizes were estimated by subtraction of fixed bicarbonate from total bicarbonate in the samples. Amounts of bicarbonate were calculated from counts per million (cpm) and the specific activity of the radioactive bicarbonate solution determined with a liquid scintillation analyser (Perkin-Elmer TriCarb 2810 TR).

## Results

### *The* cia8 *mutant requires high CO_2_ conditions to grow well*

**Fig. 1.**
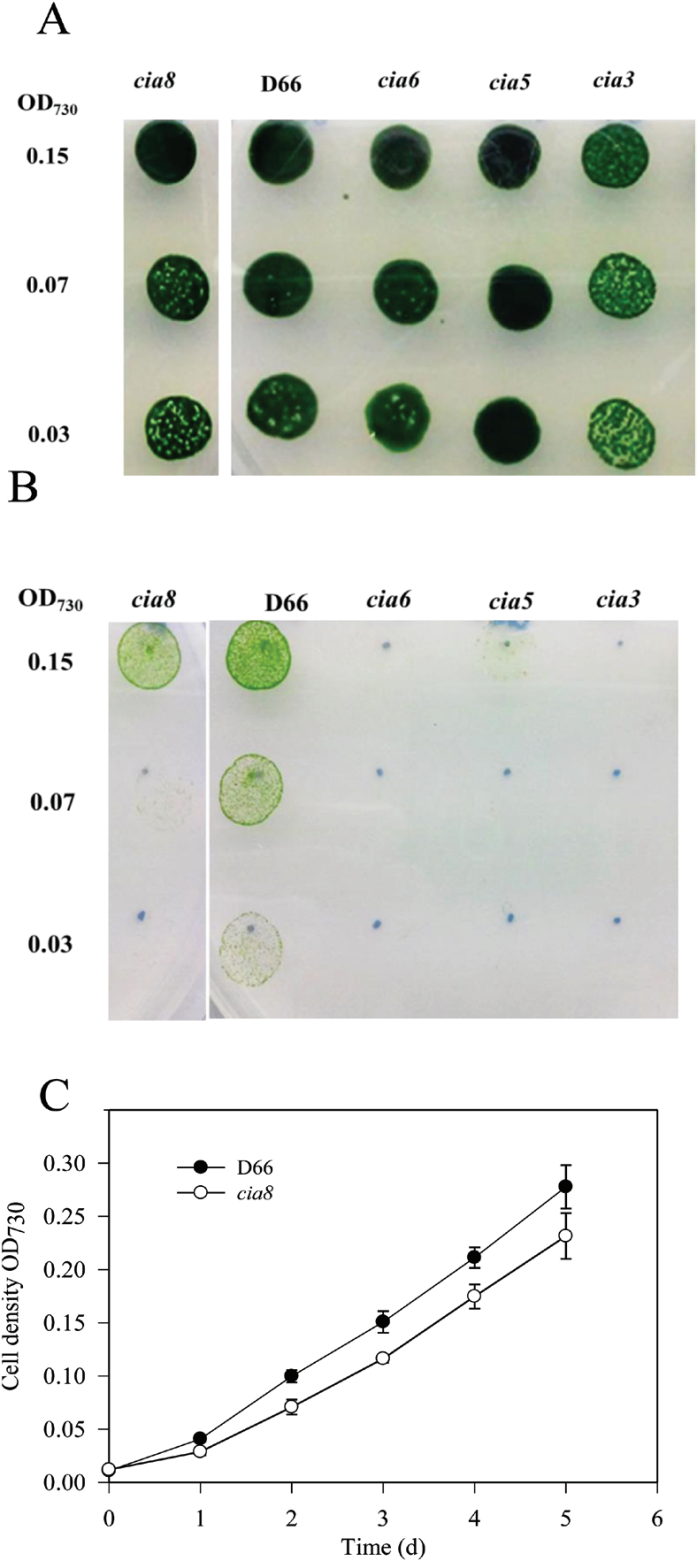
Spot test for the growth of *C. reinhardtii* strains in (A) high CO_2_ (5% CO_2_ in air) and (B) low CO_2_ (0.01% CO_2_ in air) pH 7.3. The strains include wild-type D66, the c*ia8* mutant, and three known CCM mutants c*ia6*, *cia5*, and c*ia3* used as controls. The numbers to the left represent the initial OD_730_ of 0.15 (~1.5 × 10^6^ cells) and two serial dilutions. (C) Growth of wild-type strain D66 and the *cia8* mutant in liquid culture (MIN). Cultures were grown in Erlenmeyer flasks blowing in low CO_2_ (0.01–0.025%). Values are expressed as the mean ± SE (*n*=4). (This figure is available in colour at *JXB* online.)

The *cia8* mutant was identified following an insertional mutagenesis screen using a 1.8-kb *AphVIII* para^R^ cassette containing the aminoglycoside 3'-phosphotransferase type VIII-encoding gene (*AphVIII*) from *Streptomyces rimosus* which confers paromomycin resistance (Sizova *et al.*, 2001). Mutants were screened by growing them on high CO_2_ (5%) and low CO_2_ (0.01%) on MIN plates in which potential CCM mutants would show slow growth when grown on low CO_2_ (Jungnick *et al.*, 2014). While the c*ia8* mutant grew well in high CO_2_ conditions (Fig. 1A), it showed impaired growth in low CO_2_ (0.01–0.025% v/v CO_2_ in air) (Fig. 1B) and was thus taken for further investigation. The slow growing phenotype was also apparent when cells were grown in liquid (MIN) culture at 0.01% CO_2_, where the doubling time for the *cia8* mutant was 30 h compared with 20 h for D66 (Fig. 1C).

### *Identification of the* Cia8 *gene and confirmation of insertional inactivation*

**Fig. 2.**
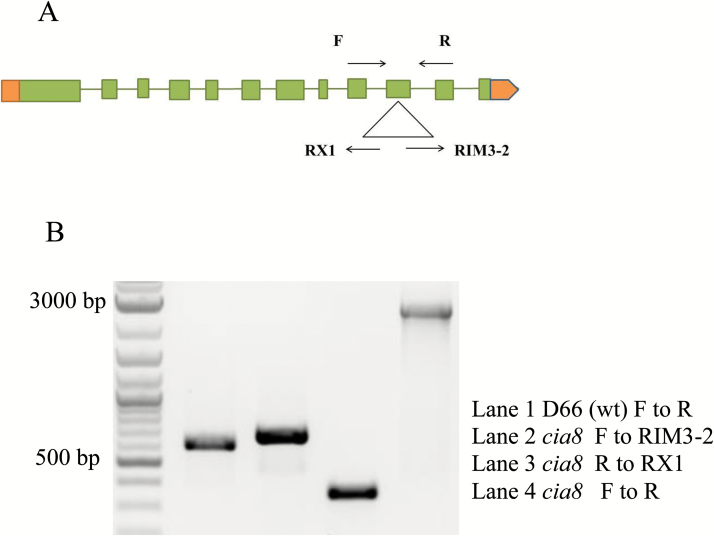
(A) Genomic structure of the *Cia8* gene locus (ID: Cre09.g395700) showing the position of the *AphVIII* insertion (triangle). Note that the cassette is in reverse orientation in relation to the direction of the gene. Green bars, spaces, and orange bars represent exons, introns, and untranslated regions, respectively. The arrows represent the direction of the primers. (B) Confirmation of *AphVIII* insertion in exon 10 of the *Cia8* gene. Lane 1: D66 is the control genomic DNA without the cassette and the other three are *Cia8* genomic DNA. Lane 2 represents the 3' end of insertion and lane 3 represents the 5' end of insertion. Lane 4 spans the whole insertion region including the genomic region flanking the cassette using a primer pair in the gene. (This figure is available in colour at *JXB* online.)

The location of the *AphVIII* insert in the *Cia8* gene was determined using the adaptor PCR described by Pollock *et al.* (2017). The insertion is located in the 10th exon of the *Cia8* gene as shown in the gene model in Fig. 2A. PCR amplification was done to confirm the presence of the insertion using two primer pairs that amplify the 5' and 3' ends of the insertion (Fig. 2B). Supplementary Table S1 has the list of primers used in the study. The genomic DNA flanking the insertion site in this mutant was isolated and sequenced. Analysis of the sequenced genomic DNA revealed a deletion of 16 bases of the gene sequence at the 5' end of the insertion, and a new region of 19 bases was inserted. However, the 3' end of the insertion was intact with no deletion or insertion. The analysis confirmed that the target gene sequence had been disrupted in the 10th exon and that no other large DNA deletions or insertions had occurred.

**Fig. 3.**
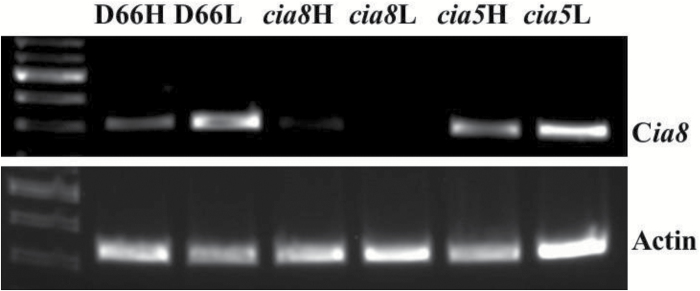
RT–PCR analysis of D66 and *cia8* mutant strains using poly(A) RNA from high and low CO_2_-grown cells as templates for RT–PCR. For the c*ia8* mutant, the primers shown in Supplementary Table S1 amplify a 1500 bp product from cDNA. GAPDH was used as a loading control and a 1000 bp product was amplified.

Disruption of transcription of the gene was also confirmed by reverse transcription–PCR (RT–PCR) using a gene-specific primer pair spanning the insertion site. RT–PCR results show that there is no detectable expression of the intact *Cia8* transcript in the c*ia8* mutant (Fig. 3), confirming that the transcript has been disrupted and that the gene product in the mutant is below detectable levels. As expected, mRNA for *Cia8* was detected in wild-type high CO_2_ cells. There was, however, a marked up-regulation of transcript in low CO_2_ cells (Fig. 3), suggesting that the *Cia8* gene is inducible under low CO_2_ conditions. The result from this RT–PCR also shows that the gene encoding CIA8 does not appear to be regulated by the *Cia5* gene, the master regulator of many CCM genes, as the transcript was expressed in the c*ia5* mutant equally to that in the wild type.

Genetic crosses of the c*ia8* mutant with the wild-type strain demonstrated a 1:1 segregation ratio of paromomycin-resistant to paromomycin-sensitive progeny, illustrating that the mutant carries a single insertion (data not shown). Further investigation by PCR confirmed that the paromomycin-resistant strains carried the cassette (data not shown). Phenotypic analysis (data not shown) showed that the tetrads with resistance to paromomycin consistently demonstrated impaired growth in low CO_2_ conditions compared with the wild type. Taking the results together, we concluded that the *Cia8* gene locus is segregating with, and therefore responsible for, the observed slow growth phenotype under low CO_2_ conditions.

### *Prediction of structure and transmembrane domains of the* Cia8 *gene*

**Fig. 4.**
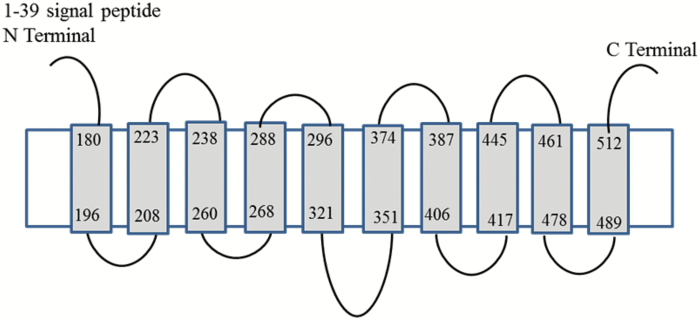
Topology model predictions for CIA8 based on the alignment with *Yersinia frederiksenii*. The grey boxes are putative membrane-spanning helices connected by loops of variable lengths; 62% of the residues were modelled at a >90% confidence level. Predictions were generated by PHYRE2 (Kelley *et al.*, 2015).

A comparison of cDNA and genomic sequences shows that *Cia8*, located on chromosome 9 in the genome, has 12 exons. On the basis of nucleotide sequence, *Cia8* is predicted to encode a polypeptide of 529 amino acids and aligns best with the sodium/bile acid (SBF-like) solute carrier protein group. We used the PHYRE2 software (Kelley *et al.*, 2015) to thread CIA8 to known protein structures. Using the core transmembrane part of the protein containing 370 amino acids (amino acids 160–529) gave the highest homology scores, and was predicted to have 10 putative transmembrane domains of 15–24 amino acids and relatively small extracellular loops between the transmembrane helices of 8–30 amino acids, as shown in Fig. 4. The protein threaded best to the crystal structure for a bacterial (*Yersinia frederikseni*) Na^+^/metabolite symporter (82%), but it also threaded well to HapA proteins (Na^+^/H^+^ antiporters) from several bacteria (56–98%).

### Similarity of CIA8 to other anion transporters

Searches in the *C. reinhardtii* protein database revealed eight other predicted proteins similar to CIA8: Cre02.g095085; Cre02.g095086; Cre02.g147450; Cre06.g250450; Cre09.g393250; Cre10.g448350; Cre12.g521950; and Cre12.g532500, all annotated as Na^+^/bile acid transporters. Sequence identity of these proteins to CIA8 varies between 19% and 31%. The number of Na^+^/bile acid transporter genes in *C. reinhardtii* is quite comparable with other species, with six having been reported in Arabidopsis (Sawada *et al.*, 2009), seven in humans (He *et al.*, 2009), and five in the marine diatom *Phaeodactylum tricornutum* (Ashworth *et al.*, 2016). The numbers are not surprising considering the variety of ions that are transported by these types of transporter proteins. The best alignment using the core (amino acids 160–529) were to chloroplast BASS4 proteins (35–40% identity with >90% coverage). The CIA8 protein also shows considerable sequence similarity to several SBF proteins from higher plant species, many of which have not yet been characterized (Supplementary Fig. S1). The closest algal homologue was found in *Volvox carteri* with 76% identity. Our analysis suggests that the *Cia8* gene product belongs to a family of conserved membrane transport proteins characterized by conserved domains.

### CIA8 has a reduced affinity for Ci

**Fig. 5.**
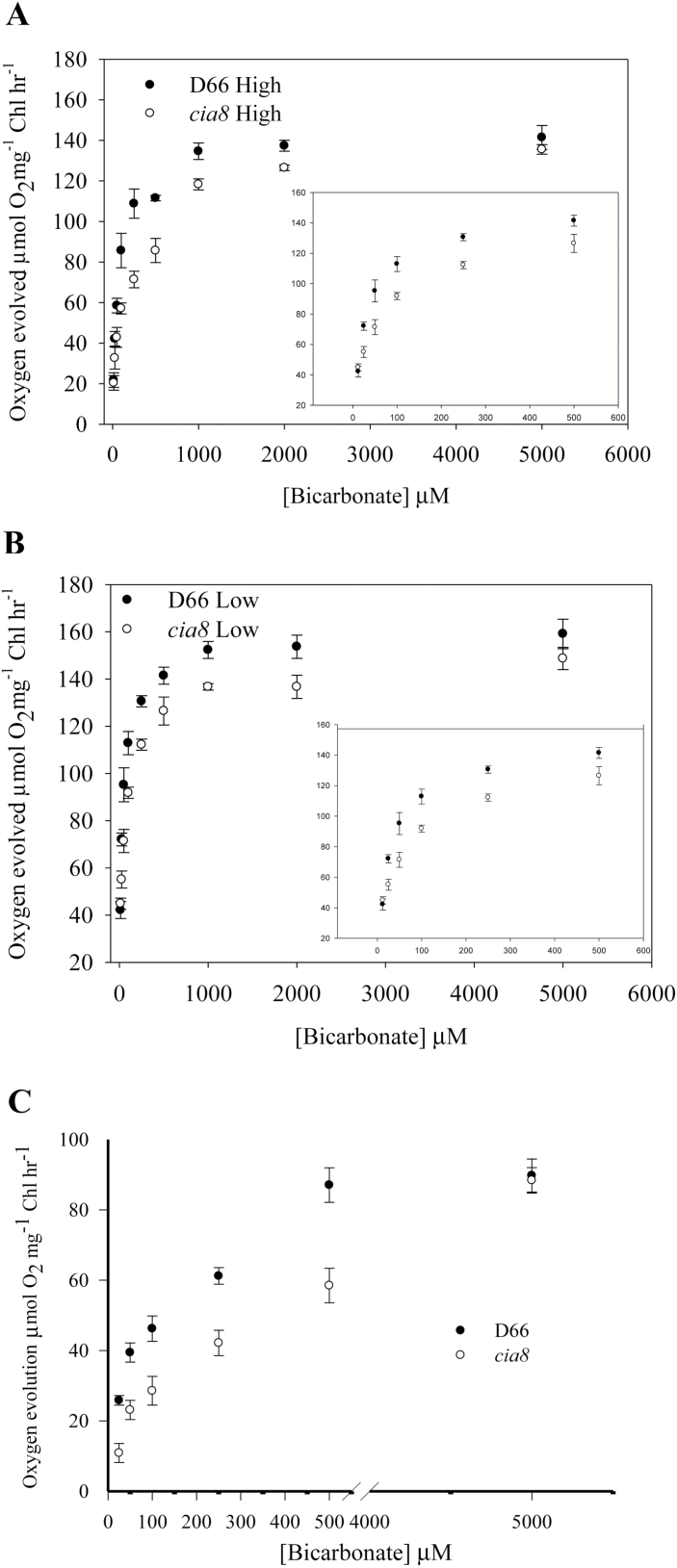
Ci-dependent photosynthetic oxygen evolution in the *C. reinhardtii* wild type and the c*ia8* mutant. (A) High CO_2_-grown cells and (B) low CO_2_-grown cells assayed at pH 7.3; (C) low CO_2_-grown cells assayed at pH 9.0. Cells were grown at high CO_2_ and transferred to low CO_2_ for 4 h. The insert in (A) and (B) represents lower concentrations >500 μM used for determination of *K*_(0.5)_. Each point represents the mean and SE of three separate experiments.

Since the expression of CIA8 is strongly responsive to CO_2_ limitation, we evaluated its possible function in the CCM using physiological assays. The strain missing CIA8 exhibited a higher *K*_(0.5)_ (Ci), indicating that they had a lower affinity for inorganic carbon than wild-type cells (Fig. 5A, B). At pH 7.3, the *K*_(0.5)_ (Ci) for wild-type cells was 25 μM. In contrast, c*ia8* mutant cells had a significantly higher *K*_(0.5)_ (Ci) of 90 μM (Table 1), suggesting a reduced affinity for Ci in the mutant. Oxygen evolution was also measured at pH 9.0 where the predominant Ci species in the medium would be bicarbonate (87% HCO_3_^–^, 13% CO_3_^2–^, <0.15% CO_2_), hence, oxygen evolution would be highly dependent on active bicarbonate uptake (Duanmu *et al.*, 2009). The shift in the *K*_(0.5)_ was also significant at higher pH, with *cia8* mutant cells exhibiting a greater *K*_(0.5)_ (Ci) of 350 μM as compared with 100 μM in the wild-type cells (Fig. 5C; Table 1). Thus, the reduced affinity for Ci is observed at both pH values. This reduced affinity for Ci in the mutant is consistent with the hypothesis that the *Cia8* gene product is important in Ci uptake.

**Table 1.** Maximal oxygen evolution activity (V _max_) and Ci affinity, K _(0.5)_ values for wild-type D66 and cia8 mutant cells Cells were grown on high CO_2_ for 48 h and acclimated to low CO_2_ for 4 h.

*V*_ max _ (μmol O _ 2 _ mg Chl ^ –1 ^ h ^ –1 ^)			*K*_ (0.5) _ Ci (μM)	
	D66	*Cia8*	D66	*Cia8*
pH 7.3 high CO_2_ cells	166 (±3.3)	155 (±4.5)	95(±2.8)	158 (±2.9)
pH 7.3 low CO_2_ cells	160 (±7.8)	150 (±5.3)	20 (±2.7)	60 (±4.7)
pH 9.0 low CO_2_ cells	92.7 (±1.5)	90 (±5.2)	110 (±5.0)	320 (±10.5)

For *V*_max_, all means are not significantly different *P*>0.1 (α=0.05). For *K*_(0.5)_ Ci, the means are significantly different from each other *P*<0.001 (α=0.05). In parenthesis, ±SEM; *n*=3.

### *Localization of CIA8 to the chloroplast in* C. reinhardtii

Analysis using YLoc (https://omictools.com/yloc-tool) (Briesemeister *et al.*, 2010) suggested that the CIA8 peptide is a chloroplast membrane protein (score 81%). Another alignment of the N-terminal sequence of this protein was conducted using SCLpred - Bologna Biocomputing Group (schloro.biocomp.unibo.it). The software predicts that CIA8 has a chloroplast transit peptide (cTP; score 0.79) and thylakoid (0.81). It also predicts a location as thylakoid membrane (0.6). As a control, we used Kea3.3, an Arabidopsis thylakoid antiporter, and the software predicts a cTP (score 0.77) and a location as thylakoid membrane (0.51). These predictions give a strong indication that CIA8 is a chloroplast protein. To confirm this subcellular localization, a *C. reinhardtii* line expressing the *Cia8* coding sequence fused to GFP (*Cia8*:CrGFP) under the control of the PsaD promoter was generated. The PsaD promoter is a high expression promoter which drives a nuclear gene encoding an abundant chloroplast protein located on the stromal side of PSI in *C. reinhardtii* (Fischer and Rochaix, 2001). RT–PCR analysis revealed that GFP transcript was detectable in this strain (Supplementary Fig. S2A). Although green fluorescence of the fused protein CIA8:CrGFP was detected in chloroplasts by live imaging on the confocal microscope (Supplementary Fig. S2B), the signal was weak and not sufficient to conclude chloroplast localization. The weak signal however, did not seem to be confined to the chloroplast envelope, but diffused throughout the organelle, suggesting a thylakoid localization.

### CIA8 transport is not dependent on Na^+^

Several solute transporters are known to be Na^+^ dependent, including BicA, a low affinity bicarbonate transporter in cyanobacteria (Price *et al.*, 2004; Price and Howitt, 2011). To determine whether *Cia8* gene function could be dependent on Na^+^ ions, wild-type and mutant cells were grown on different concentrations of Na^+^ (<10 µM to 200 mM). Both cultures grew well on low Na^+^ concentrations (<10 µM) up to 100 mM on pH 6.8 plates (Supplementary Fig. S3). Growth of cells was, however, inhibited at 200 mM Na^+^ (data not shown), probably due to osmotic stress. The observation that wild-type and *cia8* cells grew well at low Na^+^ concentrations (<10 µM) may imply that the Na^+^ ions are not required for the acquisition of HCO_3_^–^ in *C. reinhardtii.* This was not an unexpected result since the external Na^+^ concentration in fresh water is generally low (<1 mM) (Pootakham *et al.*, 2010). However, since some of the transporters, including CIA8, are internal, it is not clear that changing the external Na^+^ concentration would alter the internal concentration. Therefore, further analysis may be required to elucidate the role. if any, of Na^+^ in Ci uptake by *C. reinhardtii* cells.

### *Expression of* Cia8 *is up-regulated on low CO_2_ conditions*

**Fig. 6.**
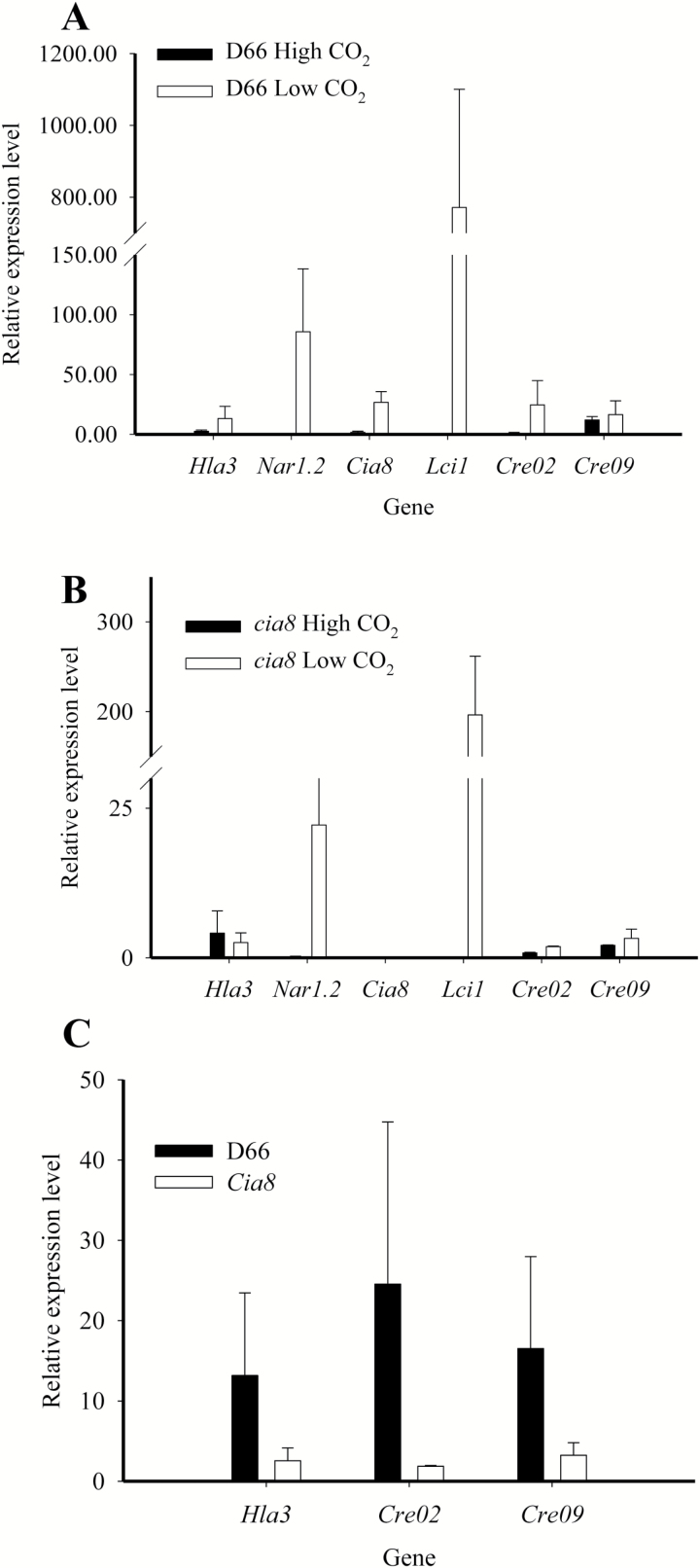
Quantitative real-time PCR results for transporter genes under high and low CO_2_ conditions in the wild-type (A) and *cia8* mutant (B) strains. Wild-type D66 and mutant cells were grown on high CO_2_ for 48 h then subjected to low CO_2_ acclimation for 4 h. Lack of the *Cia8* gene causes down-regulation of other CCM transporters and two other genes in the SBF family (C). The *Cblp* gene was used as the internal control. Relative expression level is expressed as 1000/2^∆Ct^ where ∆Ct=Ct_gene_–Ct_cblp._

To quantify and compare the expression of the other known transporters in *Chlamydomonas* in the c*ia8* mutant and in the wild type, we investigated the expression of *Hla3*, *Nar1.2*, *Lci1*, and *Cia8* as well as two other SBF genes (Gene ID: Cre02.g147450 and Cre09.g393250) by qPCR analysis. RNA samples were obtained from high CO_2_- and low CO_2_-acclimated cultures. The results show the level of expression of these transporter genes in the wild type (Fig. 6A) and in the *cia8* mutant (Fig. 6B) relative to the *Cplb* gene which encodes a G-protein beta subunit-like polypeptide and also known to exhibit constitutive expression of transcript (Schloss, 1990). The results confirm up-regulation of the *Cia8* gene and the other transporters upon acclimation to low CO_2_ conditions, except for *Hla3* which usually increases in expression at times >4 h (Duanmu *et al.*, 2009). In the wild type, there was a 4-fold up-regulation of the *Cia8* gene on acclimation to low CO_2_ conditions. There was also a notable up-regulation of the two SBF genes in the wild-type cells. The relative expression levels of these two SBF genes were also somewhat lower in the *cia8* mutant cells as compared with the wild type (Fig. 6C). While the data confirm that the *Cia8* gene product is involved in the CCM, they also suggest that loss of CIA8 may affect the transcriptional control of other genes normally up-regulated under low CO_2_ conditions.

### *The* Cia8 *gene can be complemented*

**Fig. 7.**
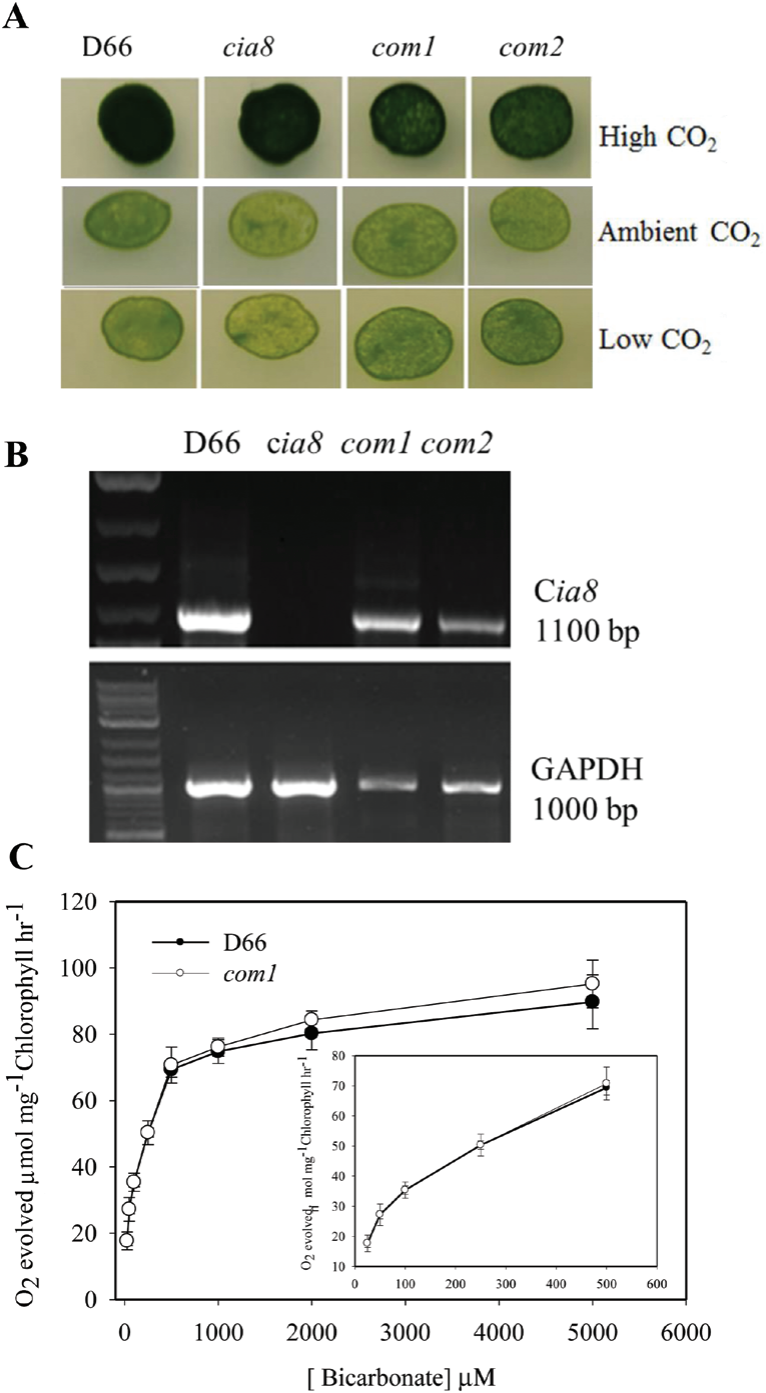
(A) Growth phenotype of complemented c*ia8* strains, *com1* and *com2*. These strains had the wild-type *Cia8* gene reintroduced into the c*ia8* mutant as described in the Materials and methods. (B) RT–PCR showing restoration of the *Cia8* transcript in the complemented strains. GAPDH was used as the loading control. (C) Oxygen evolution for the wild type, D66, and one complemented line, *com1*, at pH 9.0. Each point represents the mean and SE of three separate experiments. (This figure is available in colour at *JXB* online.)

**Fig. 8.**
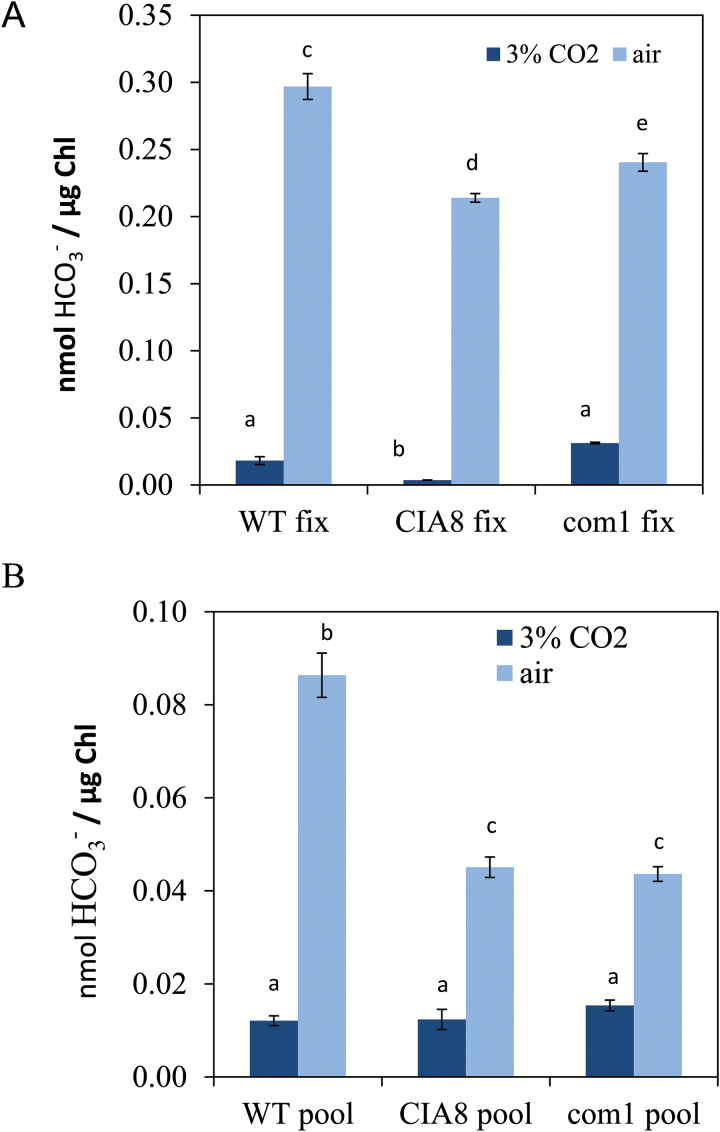
Accumulation of ^14^C in wild-type (WT) D66, in the *cia8* mutant, and in the complemented cell line *com1*. Cells were grown on elevated CO_2_ (5% v/v CO_2_ in air) and acclimated to low CO_2_. Analysis was done at pH 9.0. (A) CO_2_ fixed and (B) CO_2_ remaining in the pool. Statistical analysis was done by Tukey’s HSD test. Different letters indicate that means are significantly different at *P*=0.01. (This figure is available in colour at *JXB* online.)

Complementation of CIA8 was achieved by expressing *Cia8* cDNA (1869 bp) including the entire 5' UTR and 3' UTR in the pSP124 vector under the control of its own promoter and terminator in the c*ia8* mutant. Transformed cells were selected on bleomycin. Ten transformants were selected and subjected to further growth tests and photosynthesis assays. Results of growth experiments show that the wild-type phenotype in low CO_2_ conditions was restored in complemented lines (referred to as *com1* and *com2*) (Fig. 7A). RNA was extracted from these two complemented strains and cDNA synthesized. The resulting RT–PCR analysis showed that *Cia8* transcript had been restored, although to levels slightly lower than the wild type (Fig. 7B). In addition, the Ci-dependent oxygen evolution assay of *com1* at pH 9.0 showed that the *K*_(0.5)_ (Ci) in the complemented strain was the same as that of the wild type. Also, the maximal oxygen evolved (*V*_max_) as well as CO_2_ fixed were not significantly different from the wild type (Figs. 7C and 8, respectively). These results collectively confirm that this Cre09.g395700 coding sequence complemented growth of the c*ia8* mutant, restoring the expression of transcript and functionality in Ci uptake.

### Ci uptake assays

To evaluate the contribution of CIA8 to Ci uptake activity, the silicone oil layer centrifugation method was used to measure accumulation of ^14^C in D66, in CIA8, and in the complemented cell line *com1*. Analysis was done at pH 9.0 to ensure the predominant Ci species was bicarbonate, using cells grown in elevated CO_2_ (3% v/v CO_2_ in air) or in air (low CO_2_) cells. In all cases, the cells acclimated to low CO_2_ had greater amounts of accumulated Ci in the cells than cells grown on elevated CO_2_ (Fig. 8). When Ci uptake and bicarbonate pools were compared, wild-type cells had greater Ci accumulation and greater Ci fixation than c*ia8* cells (Fig. 8). In addition, photosynthesis was nearly restored to wild-type levels in the *com1* cells line, although Ci accumulation was not fully restored. These data support the hypothesis that Ci uptake, most probably in the form of bicarbonate, is reduced in *cia8* cells.

## Discussion

The need for the uptake and accumulation of bicarbonate is a key feature of the *C. reinhardtii* CCM. The putative bicarbonate transporters in *C. reinhardtii* are generally induced by limiting CO_2_ conditions. It also appears that the transporters have overlapping partially redundant functions. If the expression of only one transporter is reduced, there usually is not a strong growth phenotype, and a small effect on Ci uptake is observed. However, if two or more transporters are reduced, Ci uptake is affected and growth on low CO_2_ is reduced (Duanmu *et al.*, 2009; Yamano *et al.*, 2015). In this work, we generated new mutants by insertional mutagenesis and identified a mutant with an insertion in the gene (Phytozome ID: Cre09.g395700) which we have named C*ia8* (for Ci accumulation). The main objective of this work was to determine whether the CIA8 protein participated in the CCM of *C. reinhardtii*. We characterized and demonstrated that the insertion in this gene caused poorer growth on low CO_2_ and resulted in a significant decrease in Ci affinity when cells were grown under limiting CO_2_ conditions. In complementation experiments, the expression of the *Cia8* coding region from the wild-type gene was able to restore the normal growth phenotype and function in photosynthetic assays (Fig. 7).

The CIA8 protein belongs to the SBF/BASS subfamily which includes membrane-bound transporters designated as bile acid transporters. These proteins transport a diverse number of substrates. In Arabidopsis, two of these genes have been characterized, and BASS2 is involved in pyruvate transport across the chloroplast envelope in the methylerythritol 4-phosphate (MEP) pathway, whereas BASS4 transports 2-keto acids during glucosinolate metabolism (Gigolashvili *et al.*, 2009; Furumoto *et al.*, 2011). Bioinformatic searches in the PFAM database reveal that some SBF and SBF-like anion transporters may be involved in bicarbonate uptake. Despite being important transporters in other eukaryotic species, this subfamily of SBF/BASS gene products remains of unknown function in *C. reinhardtii*.

Physiological characterization of CIA8 in this study supports the idea that it is involved in CCM function and Ci uptake under limiting CO_2_ conditions. The CIA8 transporter seems to make a significant contribution to CCM function because it is the only transporter so far that shows a distinct phenotype in single knockout mutants (Fig. 1). The other known transporters HLA3 and NAR1.2 did not show a definitive growth phenotype as single knockout mutants (Yamano *et al.*, 2015), and no growth differences were reported when LCI1 was expressed in the *Lcr1* background (Ohnishi *et al.*, 2010). In this study, the apparent affinity of mutant and wild-type cells for Ci at both pH 7.3 and 9.0 was tested. At pH 9.0, most of the Ci is in the form of bicarbonate. Under these conditions, a significant fraction of the Ci is thought to be transported as HCO_3_^–^ at both the plasma membrane and the chloroplast envelope (Wang *et al.*, 2014). The loss of CIA8 clearly reduces the apparent affinity of the cells for Ci at both pH values. The decreased rate of Ci-dependent photosynthesis and reduced affinity for Ci in the c*ia8* mutant at pH 7.3 and 9.0 (Fig. 5) provides evidence that CIA8 might be involved directly or indirectly in Ci uptake.

While SBF transporters are often associated with co-transport of Na^+^ ions as revealed in some animal proteins (Romero *et al.*, 2013), the results in this study suggest that Na^+^ ions may not necessarily be essential for the functionality of the CIA8 protein (Supplementary Fig. S3). Low Na^+^ ion concentrations (i.e. <10 µM to 100 mM) did not affect the growth of cells in the mutant and wild type. This was not a surprising result since TAP and MIN media in which *C. reinhardtii* cultures are grown contain no added sodium. Thus, *C. reinhardtii* cells grow well with very low concentrations of sodium. While there is ample support for Na^+^-coupled transport in marine algae and freshwater cyanobacteria (Shibata *et al.*, 2002; Price *et al.*, 2004; Nakajima *et al.*, 2013), H^+^-coupled transport has always been thought to play a role in freshwater algae. However, other ions such as Na^+^ or K^+^ might be coupled to bicarbonate movement. Another possibility could involve an exchange (antiport) of bicarbonate for an anion such as chloride.

Our analysis of the gene transcripts showed that the c*ia8* mutant is a knockout. In the wild type, the *Cia8* transcript was strongly up-regulated (4-fold) when high-CO_2_ cells were acclimated to low CO_2_, and this induction is consistent with the other Ci transporters (Figs 3, 6) although the level of induction is not as high as some of the CCM proteins. Regulation of transporters by transcript abundance in *C. reinhardtii* is well documented (Ohnishi *et al.*, 2010; Gao *et al.*, 2015; Yamano *et al.*, 2015). This type of regulation arguably prevents expression of HCO_3_^–^ transporters under high CO_2_ conditions where they would not be required. It is interesting that the *Cia8* gene is expressed, albeit at a low level, in high CO_2_ conditions, which may suggest a housekeeping function for the protein. Additionally, the relative expression of the other transporters is down-regulated in the c*ia8* mutant as compared with the wild type (Fig. 6). This could be explained by a negative feedback mechanism whereby the loss of CIA8, a putative chloroplast envelope protein, leads to accumulation of HCO_3_^–^ in the cytosol, which negatively impacts the two plasma membrane transporters (HLA3 and LCI1), and later NAR1.2. Alternatively, it may be that *Cia8* gene may have some form of regulatory control over these other genes, just like the *Nar1.2* gene transcript which has recently been shown to regulate the expression of *Hla3* (Yamano *et al.*, 2015). However, the transcriptional changes we observed were relatively small and could simply reflect the fact that the *cia8* cells are growing more slowly at the lower CO_2_ concentration. These results raise the possibility that the contribution of CIA8 to CCM function may thus be more than simply Ci uptake, an interesting aspect that requires further investigation.

Several studies in the past have hypothesized the occurrence of a putative HCO_3_^–^ channel or transporter on the thylakoid membrane of the algal CCM (Raven, 1997; Wang *et al.*, 2011; Jungnick *et al.*, 2014). In this study, the signal of the GFP fused to CIA8 was too weak to provide a conclusive result, but seemed to be evenly diffused throughout the chloroplast (Supplementary Fig. S3). We speculate that the weak CrGFP signal may be attributed to a general low expression, poor folding, or poor targeting of this protein. It is also possible that the CIA8:CrGFP chimeric protein is not highly stable and is degraded. Nevertheless, the prediction programs did provide strong evidence for this localization to the organelle. Localization throughout the organelle, however, would suggest that the protein may be on the thylakoid membrane, a strategic location to pump HCO_3_^–^ directly into the thylakoid lumen. At this point, it is not clear whether CIA8 is a symporter or antiporter. However, if it is on the thylakoid membrane it might transport an ion using the transmembrane H^+^ gradient set up in the light. If CIA8 is on the thylakoid membrane, the NAR1.2 protein localized on the chloroplast envelope might be unable to make up for the loss of CIA8. We expect that future analyses of Ci uptake in a double knockout C*ia8/Nar1.2* mutant will be very informative. Also with this potential thylakoid localization, the *Cia8* gene product could be making a significant contribution to CCM function, and perhaps to Ci uptake, possibly in conjunction with another protein with a function similar to CIA8.

In conclusion, the CIA8 protein is a new putative transporter that plays a role in the *C. reinhardtii* CCM. When the gene is knocked out, the mutant shows a compromised Ci uptake and growth inhibition when grown in limiting CO_2_ conditions. We propose that the *Cia8* gene encodes a putative bicarbonate transporter that is involved in uptake of HCO_3_^–^ in the chloroplast for CO_2_ fixation. The predicted CIA8 protein is hydrophobic and, according to prediction programs, has a topology comparable with other known transporters in the SBF/BASS subfamily. Further studies need to be carried out to quantify the contribution of the protein to Ci uptake in *C. reinhardtii* cells. The function of the CIA8 homologues in *C. reinhardtii* also remains to be determined. To our knowledge, this is the first report of an SBF/BASS gene in *C. reinhardtii* being involved in CCM function, and possibly in bicarbonate uptake.

## Supplementary data

Supplementary data are available at *JXB* online.

Table S1. Sequences of primers used in this study.

Fig. S1. Clustal Omega alignment of the CIA8 primary protein sequence with other SBF transporters from higher plants and other chlorophytes.

Fig. S2. (A) RT–PCR analysis of the c*ia8* mutant strain transformed with the fused CIA8:CrGFP protein. (B) Live imaging of the CIA8:CrGFP transformant cells showing the localization of the chimeric protein

Fig. S3. Growth of wild-type D66 and *cia8* mutant cells of *C. reinhardtii* on MIN plates (pH 6.8) with <10 µM up to 100 mM NaCl.

## Supplementary Material

Supplementary Table_S1_Figures_S1_S3
