# Supplementary material for: Identification and characterization of a solute carrier, CIA8, involved in inorganic carbon acclimation in Chlamydomonas reinhardtii
Source: J Exp Bot. 2017 Jun 13;68(14):3879–90. doi: 10.1093/jxb/erx189 (PMC5853530; doi:10.1093/jxb/erx189)
Supplement: Supplementary Table_S1_Figures_S1_S3 [file erx189_suppl_supplementarytable_s1_figures_s1_s3.pdf]

Supplemental Data

Supplemental Figure S1: Clustal Omega alignment of the CIA8 primary protein sequence with other SBF transporters from higher plants and other chlorophytes. List of species and their accession numbers in NCBI or Phytozome: *Monoraphidium neglectum* XP_013904301.1; *Monoraphidium neglectum*, XP_013896513.1 *Coccomyxa subellipsoidea* C-169] XP_005649764.1 *Picea sitchensis*, ABR16540.1 *Musa acuminata* subsp. Malaccensis, XP_009404829.1 *Nelumbo nucifera*, XP_010271688.1 *Vitis vinifera*, XP_002266805.1 *Citrus sinensis*, XP_006485208.1 *Sorghum bicolor*, XP_002440278.1, *Populus euphratica* XP_011032852.1, *Volvox carteri* f. nagariensis, XP_002955170.1 *Volvox carteri*, locus Name Vocar.0008s0179; *Ostreococcus lucimarinus* locus name gwEuk.2.273.1;

Supplemental Figure S2. A. RT-PCR analysis of the c*ia8* mutant strain transformed with the fused CIA8:CrGFP protein. Analysis was done using poly (A) RNA from low CO_2 grown cells as templates for Reverse Transcriptase PCR. The primers shown in Table S1 amplify the GFP transcript (710 bp) from cDNA. B. Live imaging of the CIA8-CrGFP transformant cells showing the localization of the chimeric protein. The cells were grown in MIN and observed with a confocal microscope using different wavelengths of light. DIC: Differential Interference Contrast; GFP: fluorescing at 410-470 nm; chlorophyll auto-fluorescence at 600-700 nm.

Supplemental Figure S3: Growth of wild type D66 and *cia8* mutant cells of *C. reinhardtii* on MIN plates (pH 6.8) with <10μM up to 100 mM NaCl. 15 μL of actively growing cell culture was inoculated at each spot and grown on ambient CO_2 (0.035%) for 7 d. (OD_{730}=0.15 ~ 1.5x10^6 cells). No differences in growth were observed in either strain when Na^+ was added.

Supplemental Table S1. Sequences of primers used in this study

Primer name	Sequence 5' to 3' end
For confirmation of insert	
CIA8-F	GCACATACTATTGAGGTGCCG
CIA8-R	CGGCTGTGTTCAGTCACCT
RIM3-1	CGGTATCGGAGGAAAAGCTG
RIM3-2	TACCGGCTGTTGGACGAGTTCTTCTG
RX1	GCCCTCATAGCCCGCCAAATCAG
RX2	AAGCCGATAAACACCAGCCC
For RT-PCR	
CIA8CDS-F3	GCCTTCCATGTCGTGTACCT
CIA8CDS-F2	CAGTACCAACAGCAGCATAG
Cre09.g395700_CIA8-R2	CTAGGCCGCCTTGTTGGC
GAPDH F	ATGGCGCCAAAGAAGGTTCTTC
GAPDH R	CTACATCTTGGCCGCCACGATC
Actin-F	GCCAGAAGGACTCGTACGTT
Actin-R	CGCCAGAGTCCAGCACGATA
For construction of localization plasmids	
CIA8CDSEcoRIRnostop	GATCGAATTCACGGCCGCCTTGTTGGCTTTG
CIA8CDSNdeIF	CATGACCATATGATGTGCGCCGGCATTCGGTC
ShortGFP-F	CCAAGGGCGAGGAG
CrGFP-R	CTCTTGTACAGCTCGTCCATG

Table S1 (contd).

Primer name	Sequence 5’ to 3’ end
For construction of complementation plasmids	
CIA8transcr_R_BamHI	TAGGATCCCTTTCATAGGTTCGTGCCTCC
CIA8prom_F_SacI	CAGAGCTCCTCAGGCAAGGTGATCAAGTG
CIA8prom_R_NotI	ACTAGCGGCCGCGTCCGCATGTTGCTTATTTC
CIA8transcr_F_NotI	ACTAGCGGCCGCGTATACATGATAGCACGCCTTG
For quantitative Real time PCR	
HLA3_4,765F	GAGCGTTGTGTGCACTGTTC
HLA3_4,884R	TCTCTCTGCGGCTACATCCT
Nar1.2_1,736F	AGTTGAGGCAGGTTGAGAGC
Nar1.2_1,855R	CCCTGACCAGTTGTTGCCAT
LCI1_1,125F	TGGTTTACTGGCCGCTTCTG
LCI1_1,244R	TCACACAGCCAATCGAGGTT
CIA8_1,745F	CTGGAGTGAGGGGGCATATG
CIA8_1,867R	TTCATAGGTTCGTGCCTCCC
SLC_cre09.g393250_1,874F	CGTTACTCCCGTACTCGTACC
SLC_cre09.g393250_1,993R	GCCCACCGAACTACAGAGAG
SLC_cre02.g147450_2,777F	ATAGGCGGCAGGGAAAACTC
SLC_cre02.g147450_2,896R	TTTGTGGAATCCGCGTGACA
CBLPF	CTTCTCGCCCATGACCAC

CBLPR	CCCACCAGGTTGTTCTTCAG

CLUSTAL OMEGA (1.2.1) Multiple Sequence Alignment

```
Ostreococcus        ---------------------FLLDNFLVCGFCLALLFGLSVPAA---GKALAKVSVSGW    36
Sorghum             GK--------RAVPPSALLINFARSNFLPLALISGVILGLLDPTL---GCLAHEY-----    108
Picea               HCQPRHLTVAKVLDTVKPVLKFARTNFLPLALITGVTIGLVNPVP---GCLAQKY-----    122
Vitis               DDASQAASSGKALIWAKPLLSFVADNFLPLALVSGVALGLANPTL---GCLADRY-----    115
Citrus              RPSDQDFASSKGLNWAKPLLKIAADNFLPLALIGGVAFGFANPSL---GCLADKY-----    106
Musa                NQSSEDSVSQKASTWAEPLLDFVATNFLPLALLSGIALGLVNPTP---GCLAHKL-----    60
Nelumbo             NLHARIPDSVLLLTWVKPILSFVGSNFLPLALVSAVSVGLANPRL---GCLAHKY-----    123
Cre09.g395700       GGGAAP---SAVSTVVGWLRKLVTEQYLPLMLLAALVAAALQPSW---GLAASKT-----    206
Volvox1             SLQTSA---TTWASLVSWFRRLVAEQYLPMMLLTALLAAALQVGSRGSAMLYGNA-----    277
Volvox2             ------------------------------------------------------------    0
Coccomyxa           -----------AQTGLQKALSFIQAQFLPLALLAAMIVGYLFPGP---GLRAADA-----    47
Monoraphidium1      ---------------MPAAAAFVQAQFLPVVLVTAICVGCSFPQA---GVAVSQL-----    37
Monoraphidium2      -----------AARACAPALAFLDQHFLPLCLTSGVAAGCAFPAA---GVEAAKL-----    77


Ostreococcus        SVIQTVCVVIIFVISGATLKTEEITQALKAGRGALGYGWVAILGLTPLLGFILVRVPYKP    96
Sorghum             -SLSKFSTFGIFVMSGLTLRTKELGTALEA-WPAALYGLGSILLLTPFVSQFIMQVQFFP    166
Picea               -SLSNWSTFGIFLVSGLTLRSGEMSAAIEA-WPAGAFGLVSILLFTPFISRLVLQLKLIP    180
Vitis               -SLSKVSTFGIFIISGLMLRSGEIGAAAEA-WPVGIFGLGSILLFTPLFSRLILQFQLQP    173
Citrus              -QLSKFSTFAIFIVSGLTLRSGEIGAAAEA-WPVGIFGLFSILLFTPYFSKLILQVQLQP    164
Musa                -SLSRFSTCGIFFISGIMLHSRELGAAVEA-WPAGLFGLGSILLITPFFSRLVLQIQLTP    118
Nelumbo             -SLSKFSTFGIFFISGLMLRSGDVGAAVQA-WPAGIFGLGLILLFTPFFSRLVLQLKLVP    181
Cre09.g395700       -QLQTAVTFTIFVLQGVMLRQGEAKKALGA-AGAIAWGMASILLITPLLAPLAGALPLQP    264
Volvox1             -GLQSAVTFAVFVLRGVMLRRGEAEKALGA-KGAILWGLASILLVTPVAAPVAGALPLQP    335
Volvox2             -----------------MLRRGEAEKALGA-KGAILWGLASILLVTPVAAPVAGALPLQP    42
Coccomyxa           -GLQSLTTTGIFIISGLGLRRGEALRALSA-WGAILYGFASILFITPLAALAVLRLPLGS    105
Monoraphidium1      PNLTAFVTTAMFVISGLQLRQGEALQALKA-RGAVAFGIISILLITPLISLAVLRLPLHP    96
Monoraphidium2      -NLSTAVTFCMFVIAGVQLRQEEAFKALQA-KGALLYGLVSILFITPLISLAVLQLPLQP    135
                                      *:  :   *  *   .  :*   ** .**  .     .

Ostreococcus        IEFRYGLALFCCVPTTLTSGVTLVRNAKGNVALALMLTVSTNLIGVFTVPFYFNAVVASG    156
Sorghum             REFITGLAIFCCMPTTLSSGVILTQLVGGNSALALAMTVSSNLLGIIIVPLSLARYI---    223
Picea               QEFVTGLAMFCCMPTTLSSGVALTQVVGGNSALALSLTVASNLLGIVTVPFMLSKLV---    237
Vitis               QEFITGLAIFSCMPTTLSSGVALTQLAGGNSALALAMTVISNLLGILIVPFSISKFI---    230
Citrus              QEFVTGLALFSCMPTTLSSGVALTHLAGGNSALALAMTIISNLLGIMIVPFSISKFI---    221
Musa                HELITGLAAFCCMPTTLSSGVALTQLVGGNSALALAMTVLSNLLGILIVPFSLSKLI---    175
Nelumbo             QEFVTGLAIFSCMPTTLSSGVALTQLVGGNSPLALAMTVVSNLLGILIVPFSISKFI---    238
Cre09.g395700       PGLALGLLVFGCMPTTLSSGVALTQVLGGNTALALLLTIATNLASVFTLPFLLPWALKTT    324
Volvox1             PGLALGLLVFACMPTTLSSGVALTQVLGGNTALALLLTISTNMASVFTLPFVLPWAMKAS    395
Volvox2             PGLALGLLVFACMPTTLSSGVALTQVLGGNTALALLLTISTNMASVFTLPFVLPWAMKAS    102
Coccomyxa           PELAFGLAVFCCMPTTLSSGVSLTQAFGGNAALALLLTVGTNLVGIFTMPFMLCWLL---    162
Monoraphidium1      PELAIGLAVFCCMPTALSSGVTLTQQIGGNVALALLLTVSTNMLGVFTMPFTLPALL---    153
Monoraphidium2      RELVLGLAIFCCMPTALSAGITFTQAAGGNVAVALLLTVTSNMLGVFTMPFVLPAML---    192
                      :  **  * *:**:*::*: :.:   **  :** :*: :*: .:. :*: :   :

Ostreococcus        PR----EMASA-----VNGAANDMSTQAVKLLVKLLFTILLPIVLGKVAREMIPAVAAFA    207
Sorghum             ----------------G--TGAGVSLPTEKLFRSLVTRLLIPLIIGKVAREASKGIADFV    265
Picea               ----------------A--QGVGVSVPAGELLKSLTLMILVPLLLGKGIRNSFNGVAKFV    279
Vitis               ----------------A--DGVGVSVPTKQLLRSLVVTLLIPLILGKVLRESFKGVADFV    272
Citrus              ----------------A--AGVGISVPTKQLFKSLVLTLLIPLILGKVLRESITGLSEFV    263
Musa                ----------------G--AGAGISVPTAQLFKSLIMMLLVPLVIGKVIRDSSKSVAEYV    217
Nelumbo             ----------------A--DGVGVRIPTEQLLRSLITTLLIPLILGKVFRDFFSGVGEYV    280
Cre09.g395700       ASIGGFGAAAAVA---GGSAAAAVRLDPVPLLVQLVQCILLPTLLGAGVRGASEGLRSWV    381
Volvox1             AALGGFGACSGGGAGVGASGGLVVQLDPVPLLLQLVQCILVPACIGAGVRGVLPGLRRWV    455
Volvox2             AALGGFGACSGGGAGVGASGGLVVQLDPVPLLLQLVQCILVPACIGAGVRGVLPGLRRWV    162
Coccomyxa           ----------------GAGNS-AVSLTPGPLLRSLMRTILAPLLVGAAARAFVPGVAGQV    205
Monoraphidium1      ----------------GPALAGSVRLEPLPLLVKLVKTILVPSLVGASIRAFVPGAAAFV    197
Monoraphidium2      ----------------GGSLG-GARLEPGPLLVRLIYSVLIPTIIGAAIRSSVPGAAALA    235
                                        .         *:  *   :* *  :*   *    .    .

Ostreococcus        NARKAELTLTNNSCLIIVVWMSISKSAKELIDTNVGTIFAVLFAAVLVHVVFLAINYAAT    267
Sorghum             DRNQQGFSVGNAVLLSLVPWIQISRSRSLILSVQVEAFAAAITVGVLIHLALLAFNIAML    325
Picea               DERRELFSMINSIFLSLVPWMQVSGSRALLLTISPMNFISAIAIGMCLHFIFLSLNTVIM    339
Vitis               DKNRKLLSMISAIFLSLVPWIQVSRSRSLLLMVKPAVFLVAIGMGTVLHLVLLAFNALSI    332
Citrus              DQNRKLFSKISAIFLSLVPWMQVSRSRSLLLMVKPQVFLVAIWMGTLLHLILLAFNAFSV    323
Musa                DRNRRSFSMISAILLGLVPWMQVSRSRSLLLTVKPAIFAIAVGMGILLHFVLLAFNTIAV    277
Nelumbo             DQNRKHFSMISAILLSLVPWVQVSISSPLLLMVKPAVFLVAIGMGILLHLILLAFNSLAI    340
```

```
Cre09.g395700       DANRRTLSVISGGLLSLVPWMQVSKALAQGVTVAPAALAAAVAWSLAFHVVYLGLNCGAA    441
Volvox1             DSNRRTLSVVSGALLSLVPWMQVSKALSQGVVVAPGALAAAVSSSLVLHAAYLALNATAA    515
Volvox2             DSNRRTLSVVSGALLSLVPWMQVSKALSQGVVVAPGALAAAVSSSLVLHAAYLALNATAA    222
Coccomyxa           DKNKKALALLSACLLALVPWMQISRAVSSTVDVSLTALAKVVAAGVAVHLVYLAFNSAAV    265
Monoraphidium1      DARKKYLTYSNALLLALVPWTQISKAVAQRVPLEAGSLLVAAAAGVGVHLAFLALNIGAC    257
Monoraphidium2      DSKKRELARVSALLLGLVPWTQVSKTVAAGVVLAPGPLAVMLVAGVAVHLSYLALNTAAA    295
                    : .:  ::  .   * :* * .:* :    :      :      .  .*    *.:*

Ostreococcus        HALG-LSG---------PERVACVMMSSQKTLPVAMTIISYLPEDVFGS-GGLIAIPCIV    316
Sorghum             HILSRLGKKGDSVFAKKEYTRAVILVSSQKTLPVMITVVEQLGGALGE--SGLLVIPCVF    383
Picea               HSLSLIFGGKRSTFGKENNARAIIIVASQKTLPVMVAIVGRLGGVLGE--AGLLVIPCVA    397
Vitis               QSLSAVSGGSKSPFAKRQNTVAFLLVASQKTLPVMVAVVEQLHGTLGE--SGLLVLPCVA    390
Citrus              WSLSVISGDCQSVFAKKENTNAVVLVASQKTLPVLVAVVEQLGCAFGE--SGLLVLPCVA    381
Musa                RSLSVVSGGDQSVFSKKENLRAVIIVASQKTLPVLVAVVEQLQGALGE--AGLLVLPCVA    335
Nelumbo             HGISAISGGGKSVFSKKENARALVLVASQKTLPVLVTVVEQLGGALGE--SGLLVLPCVA    398
Cre09.g395700       T-LLRLGGSDP--VAAAATRRALIIVASQKTLPVAMAVLGRLAPAVGAEAAGCAAVTAVF    498
Volvox1             Q-VFQLGGSDP--RVAAPTRRAVVVVASQKTLPVAMAVLGRLGPVVGAEAAGCAAVTAVF    572
Volvox2             Q-VFQLGGSDP--RVAAPTRRAVVVVASQKTLPVAMAVLGRLGPVVGAEAAGCAAVTAVF    279
Coccomyxa           Q-LLRIGGPPG--KESAGERRALILVGSQKTLPIAVTVLGQLGSVLPGP-VGIAVVPCVV    321
Monoraphidium1      R-LLRLGGPDP--AAALAVRRAVILVGSVKTLPVAVSVLASLGPALGPM-AGVAVVPAMS    313
Monoraphidium2      LRLGEVA--GP--KGARDIRRAVILTASVKTLPVAVAVFASLAPVLGGM-LGVALVPALM    350
                      :  :               * :: .* ****: :::.  *   .     *   : .:

Ostreococcus        CHITQLFMDA--------------------------------------------------    326
Sorghum             AHINQIIVDSIIVNWWRRRDQQNK------------------------------------    407
Picea               AHINQIIMDSFLVNIWLQQDKRALQVKET-------------------------------    426
Vitis               AHLNQIIMDSFLINIWLGKDCT--SDNAKVA-----------------------------    419
Citrus              AHLTQIIMDSFLVNFWLRRDKDLSSNNAKVA-----------------------------    412
Musa                LHINQIIIDSFLVNWWLRRDQISAKSKEV-------------------------------    364
Nelumbo             AHINQIIIDSFLVNFWLHKDRSSNMDKEA-------------------------------    427
Cre09.g395700       SHLAQTCVDFALVSRWLEHIQRRDIKANKAA-----------------------------    529
Volvox1             SHLAQTCVDFWLVSRWLDRIRRRENQLAATRQLGSAATGLDGCATGLDGSS----C---S    625
Volvox2             SHLAQTCVDFWLVSRWLDRIRRRENQLAATRQLGSAATGLDGCATGLDGSS----C---S    332
Coccomyxa           SHLSQILIDSFLVSHWLRQDADSQQELKGRTA----------------------------    353
Monoraphidium1      AHLSQIIIDSMLVARWQAQDRAAKAA----------------------------------    339
Monoraphidium2      AHLSQILIDSAIVARWQAQARAEAAAAAAAAEAAAAAEAEAAVVAGAAEQRRLQVERDEV    410
                     *: *  :*
```

Supplemental Figure S1

A

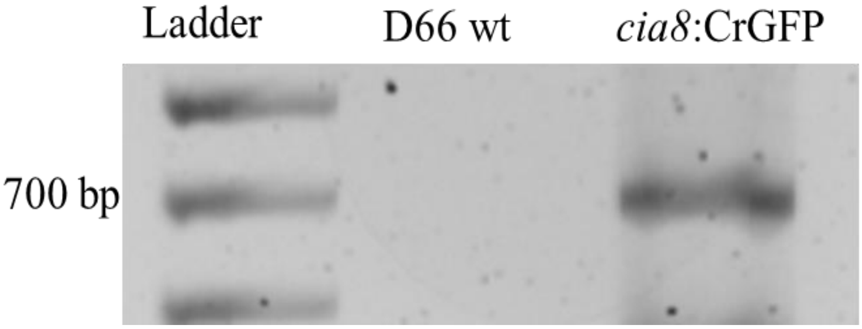

B

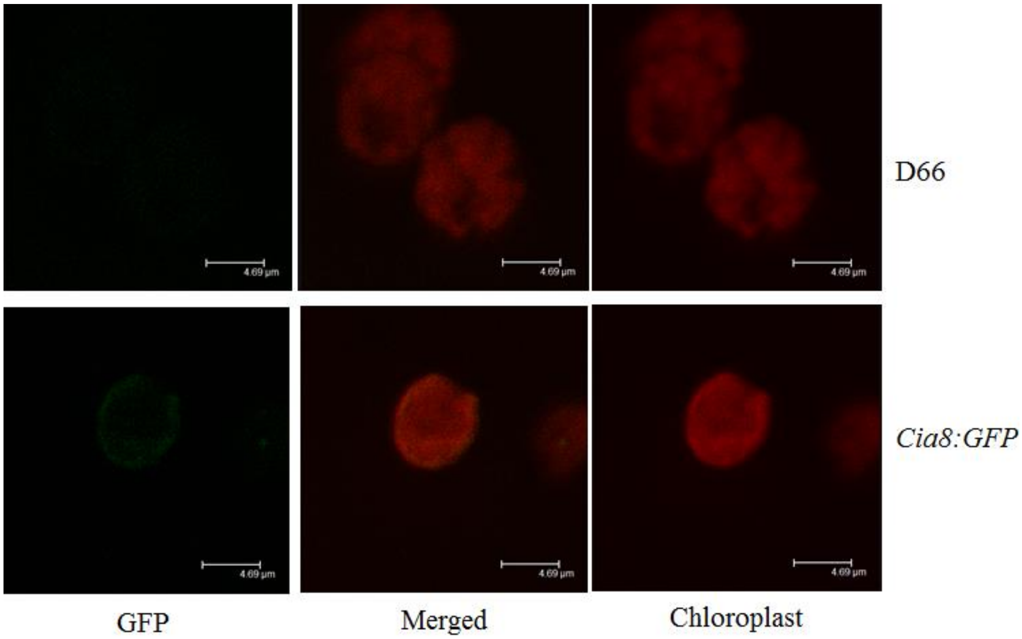

Supplemental Figure S2

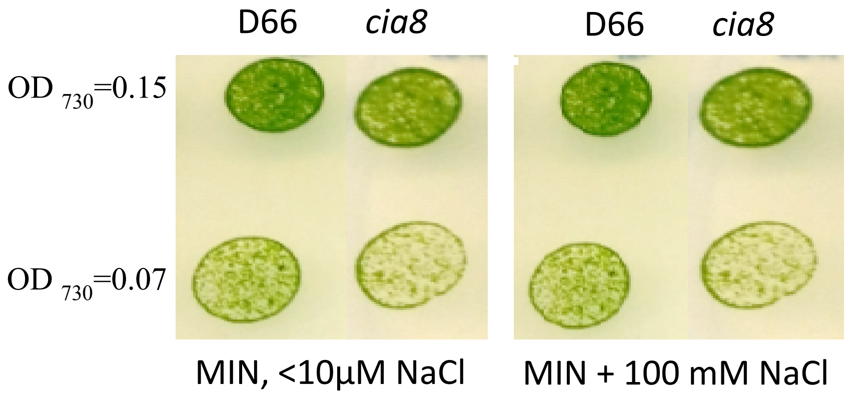

Supplemental Figure S3
